# Simulating microgravity with 60 days of 6 degree head-down tilt bed rest compromises sleep

**DOI:** 10.1038/s41526-024-00448-7

**Published:** 2024-12-05

**Authors:** Luise Strauch, Melanie von der Wiesche, Alexandra Noppe, Edwin Mulder, Iris Rieger, Daniel Aeschbach, Eva-Maria Elmenhorst

**Affiliations:** 1 Institute of Aerospace Medicine, Department of Sleep and Human Factors Research, German Aerospace Center (DLR), Cologne, Germany; 2 Institute of Aerospace Medicine, Study Team, German Aerospace Center (DLR), Cologne, Germany; 3 Institute of Aerospace Medicine, Research Relations and Development, German Aerospace Center (DLR), Cologne, Germany; 4 Institute of Experimental Epileptology and Cognition Research, University of Bonn Medical Center, Bonn, Germany; 5 Institute for Occupational, Social and Environmental Medicine, Medical Faculty, Rheinisch-Westfälische Technische Hochschule (RWTH) Aachen University, Aachen, Germany

**Keywords:** Outcomes research, Neurology, Risk factors

## Abstract

Astronauts in space often experience sleep loss. In the AGBRESA (Artificial Gravity Bed Rest) study, we examined 24 participants (mean age ± SD, 33 ± 9 years) during two months of 6^o^ head-down tilt (HDT) bed rest, which is a well-established spaceflight analogue. Polysomnography was recorded during baseline (BDC-9), HDT (nights 1, 8, 30 and 58) and recovery (R, nights 1 and 12). Mixed ANOVAs with post-hoc step-down Bonferroni adjustment indicated that compared to BDC-9, arousals were increased, while sleep duration, N3, and sleep efficiency were all decreased during HDT. Significant quadratic associations between sleep duration and quality with time into HDT did not indicate adaptive improvements during the course of HDT. While sleep duration recovered quickly after the end of bed rest, participants still displayed protracted sleep fragmentation. We conclude that physiological changes caused by exposure to microgravity may contribute to persistent sleep deficits experienced during real space missions.

## Introduction

Enormous effort is invested in the selection and training of astronauts, with the premise that optimal performance under the extreme environmental conditions of spaceflight is of utmost importance for crews’ health, safety, and wellbeing, as well as for mission success. Sufficient sleep of good quality is a prerequisite for high-level cognitive performance. The timing of sleep and waking is regulated by two processes^1^—a circadian and a homoeostatic (sleep-wake dependent) process—whose interaction grants consolidated sleep during the night, and alertness and good cognitive performance during the day^2^. However, self-reports and studies have confirmed that sleep deficiency is pervasive among astronauts^3,4^. Indeed, the average sleep duration in-flight of 6.5 h^5^ or less^3^, is below the recommended sleep duration for adults of 7 to 8 h^6^. On Earth, at least, this results in cumulative sleep and performance loss^7^. Sleep is necessary for maintaining health^8,9^, and it serves to preserve neurobehavioral functions, including learning and memory^10,11^, attention, and executive functions^7,12–14^. Even though the neurobehavioral impairment caused by sleep loss differs among individuals^15^, a general worsening of performance can be observed from the beginning to the end of a mission^16^. Especially when astronauts have to perform critical operations such as extravehicular activities, performance impairments can be very dangerous. Sleep data from nights prior to extravehicular activities indicate that sleep duration, with an average of 5.6 h, is even more reduced^4^. As an intended countermeasure, astronauts frequently take sleep medication. Use of such drugs in space is 10–20 times higher than in the general population on Earth^17^, and accounts for about 45% of all drug use by astronauts in space^3^. In a study by Wotring (2015), 71% of astronauts reported the use of sleep medication on International Space Station (ISS) missions between 2002 and 2012 that lasted an average of 159 days^18^.

Objective data on sleep during spaceflight is rare and hard to acquire, thus, sample sizes are usually small. However, the few studies that used polysomnography (the gold-standard for measuring sleep) found that sleep architecture was altered, and that N3 sleep also called slow-wave sleep, a measure of sleep quality that is important for recovery, was occasionally reduced^5,19,20^. These sleep alterations most likely have multifactorial causes. It is believed that the environmental conditions on the ISS, such as the higher noise level, artificial lighting, frequent changes of external light and dark, slam shifts (i.e. acute shift in sleep and wake times), and confinement have negative effects on sleep, but to date, it is unknown if the exposure to microgravity per se has an impact on sleep architecture. In four astronauts aboard the Russian MIR station, sleep duration and sleep efficiency seemed reduced, latency to first REM (rapid eye movement) sleep shortened, and circadian phase delayed^19^ although the results did not reach statistical significance, presumably due to the small sample size. In another study on sleep on a 10- and 16-day shuttle mission, five astronauts showed more wakefulness and less slow-wave sleep in the final third of the sleep episodes. Their sleep duration in-flight, measured with actigraphy, was significantly shorter compared to post-flight^5^. Objective data indicated even shorter than average sleep durations of 5.4 h when sleep periods took place during circadian misalignment^17^. Circadian misalignment defines a state of mismatch between the internal circadian clock and external environmental/behavioural cycles. It can result from slam shifts (as mentioned above), where a sudden change in the sleep-wake cycle forces astronauts to sleep at unusual or adverse circadian times, causing sleep disturbances^21^.

Microgravity can be simulated on Earth using a head-down tilt (HDT) bed rest regime. Long-duration HDT bed rest studies provide unique insights into changes in the physiology of bone, muscle, and the cardiovascular system associated with spaceflight^22,23^. Due to the immobilisation and inactivity during the HDT bed rest, the upward fluid shift is induced, which causes a 10–15% reduction in plasma volume. The resulting cardiovascular changes are similar to those in space. Body weight, muscle mass, and muscle strength are reduced, and the circadian rhythms may be shifted^24,25^. Boschert et al.^26^ used a 12° HDT position for one night and compared the recorded sleep to a night in the horizontal position. They found a significant increase in the percentage of light sleep (N1 + N2) and a decrease in the percentage of N3 and REM sleep. Total sleep time, however, was not affected. Participants rated their subjective sleep quality as decreased.

In comparison to Boschert et al.^26^, we are exploring the long-term effects that HDT bed rest can have on sleep duration, architecture, and quality. In the context of AGBRESA (Artificial Gravity Bed Rest Study), we aimed to evaluate the impact of simulated microgravity on polysomnographically measured sleep, self-reported sleep quality, and sleepiness. AGBRESA was a long-duration 60-day 6° HDT bed rest study initiated by the European Space Agency (ESA), the National Aeronautics and Space Administration (NASA), and the German Aerospace Centre (DLR), and hosted by the DLR’s Institute of Aerospace Medicine. In contrast to studies conducted in space, factors such as noise, high workload, lighting, and confinement played, if any, only a minor role or were kept constant, and thus changes were expected to be driven mainly by the exposure to simulated microgravity. Based on what is known from sleep in space and in simulated microgravity, we expected sleep to be shorter and to contain less N3 sleep, to be more fragmented with more arousals from sleep and awakenings during HDT. We hypothesised that participants’ self-ratings of sleep quality and feeling of recuperation after sleep would be decreased and sleepiness increased during HDT. In the recovery phase, we, on the other hand, presumed to see an improvement in objective and subjective sleep parameters due to the lack of the simulated microgravity effect.

## Methods

### Participants

The study was approved by the Ethics Committee of the North Rhine Medical Board. Participants gave written informed consent before participating in the study. The study is registered at the German Clinical Trials Register (number: DRKS00015677, registration date: 2018-10-02, https://drks.de/search/en/trial/DRKS00015677).

To participate in the study, volunteers were given detailed information about the study and had to undergo a thorough psychologic and medical screening consisting of questionnaires, an interview with two psychologists, medical anamnesis and physical examination including blood and urine testing, ECG, and ophthalmologic examination. Only healthy, non-smoking men and women with a body mass index (BMI) between 19–30 kg/m^2^ and a body height between 153–190+/−1 cm were included. A history of sleep disorders or substance abuse were further exclusion criteria. Finally, a total of 24 individuals participated in two campaigns. Demographics are detailed in Table 1. Subjects received an allowance for their participation.

**Table 1:** Participants’ demographics

	Full sample	Males	Females	Unpaired Wilcoxon
Sample size	*N* = 24	*N* = 16	*N* = 8	NA
Age (years)	33.29 (1.87)	33.06 (2.47)	33.75 (2.88)	0.6844
BMI (kg/m^2^)	24.14 (0.44)	24.37 (0.55)	23.69 (0.78)	0.6101
AHI (score)	3.30 (0.64)	4.33 (0.83)	1.25 (0.42)	0.0102
PLMI (score)	8.58 (2.87)	9.63 (3.79)	6.48 (4.34)	0.5921
T89% (%)	0.02 (0.01)	0.03 (0.02)	0.00 (0.00)	0.3781
SpO_2_ (%)	95.88 (0.20)	95.69 (0.28)	96.25 (0.16)	0.2712
PSQI (score)	3.96 (0.38)	4.25 (0.46)	3.38 (0.65)	0.2562

Mean values and standard errors (SE) are reported. The groups of female and male participants were compared with unpaired Wilcoxon tests.

*BMI* body mass index, *AHI* apnoea-hypopnea index, *PLMI* periodic leg movement index (only when accompanied by EEG arousal), *T89%* percent of sleep time spent below 90% oxygen saturation, *SpO2* mean oxygen saturation, *PSQI* Pittsburgh Sleep Quality Index.

### Protocol

**Fig. 1:**
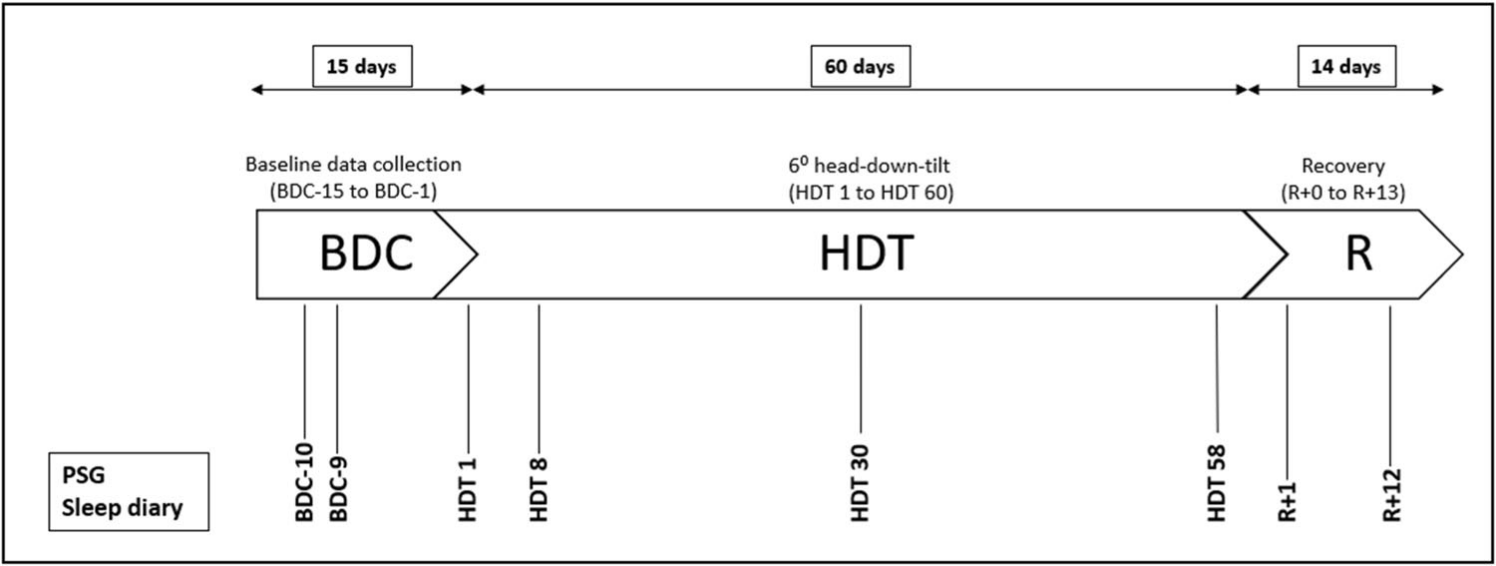
Protocol of the AGBRESA study. *BDC* baseline data collection, *HDT* head-down tilt, *R* recovery.

AGBRESA was conducted in 2019 in the :envihab facility of the DLR Institute of Aerospace Medicine in Cologne (https://www.dlr.de/envihab/en/desktopdefault.aspx/), where the participants stayed and were supervised for a total of 89 days. The study was split into two measurement campaigns with 12 participants each, and each campaign comprised a 15-day-adaption phase for baseline data collection (BDC-phase: BDC-15 to BDC-1) followed by 60 days of bed rest with 6° HDT (HDT-phase: HDT1 to HDT60), and a 14-day phase of recovery (R-phase: R + 0 to R + 13). Within each campaign, the participants were randomly assigned to three subgroups to compare the effects of continuous versus intermittent artificial gravity as a countermeasure using centrifugation on a short-arm centrifuge^27^ versus control: (1) eight participants were centrifugated continuously everyday for 30 min, (2) eight participants were intermittently centrifugated for 30 min everyday (intermittent centrifugation: 6 sequences of 5 min centrifugation with 3 min rest between exposures), and (3) eight participants served as control and did not receive any intervention. Many additional experiments were conducted during AGBRESA, such as blood-, urine-, saliva- and muscle-samples, ophthalmologic examinations, and MRI brain scans, audiometry, neurologic tests and memory tasks. During bed rest, participants remained in strict head-down tilt 24/7, which means that all procedures during day and nighttime were conducted in this position (including, e.g. eating, taking a shower or using the bedpan) and that at least one shoulder was in constant contact with the mattress or waterproof stretcher. Thorax and the head of the participants were constantly monitored via camera to ensure adherence, with the exception of bedpan and shower times. Pillows were not allowed, except for a dedicated flat pillow that was to be used in the side position only. Participants undergoing intervention were tilted to 0° bed rest on the centrifuge. For a total of eight nights, we performed polysomnographic recordings (PSG) and let the participants complete a sleep diary: ten nights prior to entry in HDT for adaption (BDC-10); nine nights prior to HDT to gather baseline sleep data (BDC-9); in the first night of HDT for acute effects (HDT1) and three more nights during the course of HDT (HDT8; HDT30; HDT58); two nights after HDT for the acute effect of recovery (R + 1) and near the end of recovery (R + 12). Fig. 1 illustrates the testing protocol.

One week prior to study entry, participants were asked to adapt their wake-sleep schedules to study-specific requirements: lights-off time (10:00–11:00 pm) and lights-on time (6:30 am), which matches the 8.5-h sleep opportunity that astronauts have at max. in space. Compliance with this request was not an exclusion criterion. Participants were also requested to refrain from caffeine, alcohol, and medication with impact on drowsiness or sleep. In-lab, participants continued to choose their lights-off time between 10:00–11:00 pm, while lights-on was scheduled at 6:30 am. Participants were free to choose light intensity according to personal preferences during wake times. Before nights of sleep recording, daytime activities were required to be as similar among participants and as little activating as possible. To offer optimal conditions for sleeping, regular sleep times and a quiet surrounding were maintained. Donning of PSG equipment was started 2 h before bedtime. Five minutes prior to bedtime and 5 min after awakening, participants filled in a sleep diary.

### Polysomnography

For the PSG recordings, electroencephalography (EEG) electrodes were attached according to standard criteria as defined by the American Academy of Sleep Medicine^28^ and the international 10–20 system (F4-A1, C4-A1, O2-A1, F3-A2, C3-A2, O1-A2; electrocardiogram (ECG), submental electromyogram (EMG), electrooculogram (EOG)). We then assessed the sleep structure after classifying EEG as either sleep stages N1–N3 and REM or awake after sleep onset (WASO) according to the criteria of the American Academy of Sleep Medicine^28^. During the first night of polysomnographic recordings (BDC-10), additional sensors (thermistor, breathing belts, EMG-electrodes at the dominant leg) were attached to validate for absence of sleep-related disorders. Sleep-related disorders were not included as part of the medical assessment but were exclusionary; the applicants provided self-disclosure of the absence of sleep disorders. In our sample, one participant had a slightly increased apnoea-hypopnea-index (AHI > 10) of 10.8 per hour of sleep, 5 participants showed periodic leg movements (PLM) > 15 per hour of sleep. However, these participants reported their sleep quality as “very good” or “rather good”, they reported no problems to stay awake or to complete everyday tasks on the Pittsburgh Sleep Quality Index during BDC. The baseline sleep assessment regarding AHI, PLMI, mean oxygen saturation, time during sleep with oxygen saturation below 90% (T89%), and PSQI is detailed in Table 1. The PSG signals were filtered (high-pass: time constant, 2.2 s for EEG and EOG; 0.04 s for EMG; low-pass: Butterworth, 12 dB/octave; −6 dB at 70 Hz for EEG, EOG, and EMG), digitised (resolution: 12-bit, sampling rate: 1024 Hz), and down-sampled for storage (256 Hz). EEG power spectra (C3-A2 or C4-A1, selected individually based on signal quality, same derivation per subject for all nights) were computed for 4-s epochs (overlap: 1 s) using a fast Fourier transformation (Hanning window). 4-s epochs with artifacts—typically arising from body or eye movements—were rejected automatically based on deviations from a moving median of EEG power in low- and/or high-frequency bands. The remaining data were averaged to yield 30-s power spectra that were matched with the sleep scores (see below). Polysomnographic EEG data from one participant were excluded due to persistent artifacts.

### Self-reports of sleep and sleepiness

Participants were asked to fill in a sleep diary 5 min before going to sleep and 5 min after lights-on, the next morning. With visual analogue scales (VASs) of 60 mm length, self-report ratings were obtained, including subjective quality of sleep (anchors “very bad” to “very good) along with the rated amount of recovery (“very bad” to “very good”), the need for sleep (“much less” to “much more”) and the feeling of sleepiness (“very sleepy” to “very much awake”). The subjective level of sleepiness was also measured using the Karolinska Sleepiness Scale (KSS). The KSS is a 9-point scale with the steps of “extremely alert” (score = 1), “alert” (3), “neither alert nor sleepy” (5), “sleepy but no difficulty remaining awake” (7), “extremely sleepy-fighting sleep” (9)^29^.

### Data reduction and statistical analysis

Data were analysed with “SAS Studio on Demand for Academics” (2021). Normal distribution of residuals was verified with Kolmogorov–Smirnov and Shapiro–Wilk tests. First, we conducted mixed analyses of variance (ANOVA, SAS: proc mixed) on all objective and self-reported sleep and sleepiness variables, including “subject” as random factor, “condition” [BDC-9; HDT1; HDT8; HDT30; HDT58; R + 1; R + 12] and “countermeasure” [continuous or intermittent centrifugation or control] as fixed factors, as well as their interaction. A significance level of 0.05 was set. Since no interactions between countermeasure and condition were found, the countermeasure subgroups were merged for all subsequent analyses. For post-hoc pairwise comparisons, we applied the Step-down Bonferroni procedure. We compared three (or four) pairs of conditions, i.e., HDT1, HDT58, R + 1 (and R + 12 provided a significant difference was present between R + 1 and BDC-9) each compared to BDC-9. Data from the adaption night (BDC-10) were not included in the statistical analyses to avoid a first-night effect^30^. For exploratory analyses of sex and age effects, we included (i) sex and the interaction sex × condition, and (ii) age and the interaction age × condition in mixed ANOVAs.

Analysis of log-transformed EEG-power spectra was conducted separately for REM and NREM (non-REM, N2 and N3) sleep. EEG power densities within 0.5 Hz bins in the range of 0.75 to 25 Hz during HDT1, HDT58, R + 1, and R + 12 were compared to BDC-9 using paired *t*-tests. The Step-down-Bonferroni procedure was used for the adjustment of post-hoc comparisons within each frequency bin.

Finally, we examined whether objective and self-reported sleep and sleepiness parameters showed continuous degradation or signs of adaptation during HDT bed rest using linear and quadratic mixed regression analyses. In case regression analyses indicated a significant effect of HDT on objective sleep parameters, we included BDC-10 values of AHI, PLMI, and T89% in the models. Results did not indicate that the effects of HDT on objective sleep parameters were impacted by AHI, PLMI, and T89%.

As objective sleep parameters, we analysed time in bed (TIB; defined as the interval between lights-off and lights-on), sleep period time (SPT; defined as the interval in minutes between sleep onset and the last epoch of sleep), total sleep time (TST; SPT minus wakefulness during SPT), sleep onset latency (SOL; first occurrence of N2 or deeper sleep), sleep efficiency (TST/Time in bed*100), number of arousals as well as arousal index (number of arousals per h sleep) and the percentage of each sleep stage N1–N3 and REM in relation to SPT, the duration of each sleep stage N1–N3, REM and wake after sleep onset (WASO). To achieve normal distribution of residuals, some of the parameters were transformed either by taking the logarithm of the parameter itself or the parameter´s highest value plus 1 minus the parameter itself (SPT; SE), or by calculating the square root (nightly arousals; SOL). Slow-wave-activity (SWA) was calculated, including frequencies from 0.75 to 4.5 Hz.

Furthermore, we converted VAS data into percentage values (e.g. a mark of 30 mm on the 60 mm scale equals 50%). From the sleep diaries, we obtained subjective SPT and SOL. Differences between BDC-9 and HDT58 were calculated for each participant and used for Pearson correlations between self-reported sleep quality as well as KSS sleepiness (morning and evening) and objective sleep parameters. Such correlations were also calculated between objective and subjective SPT and between objective and subjective SOL.

## Results

### Polysomnography

Female and male participants did not differ in demographics and at BDC-10 assessed parameters such as PLMI, T89%, and mean oxygen saturation except for AHI (Table 1).

Table 2 gives an overview on the descriptive statistics of objective sleep parameters and self-reports. Self-reports include ratings of sleepiness before each examined night as well as ratings of sleep and sleepiness after each examined night.

**Table 2:** Descriptive statistics of objective and self-reported sleep parameters

	Condition, mean (SE)						
	BDC-9	HDT1	HDT8	HDT30	HDT58	R + 1	R + 12
Objective parameters							
TIB (min)	455.12 (3.04)	445.39 (1.76)	448.02 (2.70)	448.17 (2.88)	453.31 (1.74)	457.75 (1.38)	447.42 (1.94)
SPT (min)	425.70 (5.56)	413.63 (5.30)^a^	408.77 (9.76)	413.58 (5.29)	400.78 (7.46)^a^	420.10 (5.82)	412.96 (6.94)
TST (min)	385.98 (4.96)	362.41 (5.81)^a^	376.90 (8.98)	373.65 (6.89)	360.78 (7.52)^a^	370.00 (9.01)	372.29 (7.72)
SOL (min)	26.10 (3.68)	26.85 (4.97)	34.38 (8.10)	27.71 (4.16)	41.83 (6.35)^a^	33.01 (4.76)	22.02 (2.80)
Arousals (total number)	123.10 (11.23)	116.22 (14.30)	127.13 (10.60)	110.38 (8.96)	221.88 (25.29)^a^	233.63 (19.94)^a^	221.58 (20.07)^a^
Arousal index (per h TST)	19.11 (1.77)	19.14 (2.4)	20.06 (1.56)	17.8 (1.56)	37.05 (4.19)^a^	38.02 (3.58)^a^	35.69 (3.36)^a^
WASO (min)	43.04 (5.01)	56.13 (5.66)	36.75 (5.02)	46.81 (7.34)	47.79 (7.63)	54.67 (8.50)	53.10 (7.99)
N1 (min)	20.50 (1.89)	19.43 (1.36)	18.50 (1.53)	18.60 (1.53)	26.30 (7.45)	17.70 (1.45)	16.70 (1.50)
N1% (% of SPT)	4.77 (0.31)	4.68 (0.30)	4.46 (0.33)	4.50 (0.38)	6.80 (2.11)	4.20 (0.33)	4.05 (0.34)
N2 (min)	197.20 (5.55)	196.30 (5.17)	199.40 (8.34)	192.70 (6.30)	180.00 (8.00)	187.50 (7.1)	189.00 (7.71)
N2% (% of SPT)	46.47 (1.40)	47.56 (1.32)	48.58 (1.66)	46.53 (1.32)	44.82 (1.82)	44.78 (1.77)	45.74 (1.69)
N3 (min)	83.30 (6.40)	71.28 (5.70)^a^	73.71 (6.43)	76.08 (6.47)	75.09 (6.56)	79.33 (6.75)	83.08 (6.19)
N3% (% of SPT)	19.54 (1.46)	17.14 (1.35)	18.44 (1.77)	18.49 (1.62)	18.73 (1.62)	18.76 (1.53)	20.27 (1.56)
REM (min)	84.90 (3.55)	75.40 (3.66)	85.30 (4.88)	86.30 (4.83)	78.50 (6.82)	85.50 (4.76)	83.40 (3.41)
REM% (% of SPT)	20.00 (0.84)	18.23 (0.84)	20.80 (1.11)	20.77 (1.09)	25.31 (5.11)	20.34 (1.11)	20.21 (0.74)
N3 latency (min)	17.83 (3.04)	12.83 (1.50)	14.04 (1.37)	19.13 (2.75)	13.73 (2.32)	16.04 (2.69)	15.44 (3.18)
REM latency (min)	81.65 (7.16)	69.61 (6.45)	63.96 (4.17)	59.54 (6.62)	58.15 (5.62)	78.42 (6.76)	63.44 (5.02)
Sleep efficiency (%)	84.86 (1.13)	81.44 (1.44)	84.12 (1.97)	83.46 (1.63)	77.45 (3.01)^a^	80.81 (1.92)	83.19 (1.66)
SWA (ln, μV^2^/Hz)^#^	1.48 (0.06)	1.49 (0.04)	1.49 (0.04)	1.48 (0.04)	1.53 (0.04)	1.51 (0.04)	1.51 (0.04)
Self-reported parameters							
Quality (%)	52.42 (4.45)	31.85 (4.10)^a^	36.40 (4.49)	32.19 (4.16)	30.41 (4.22)^a^	37.51 (4.84)^a^	39.03 (5.37)^a^
Recovery (%)	51.10 (4.12)	53.64 (4.68)	50.82 (5.33)	50.98 (5.37)	64.03 (4.80)	65.78 (4.11)	45.36 (5.56)
Need for more sleep (%)	68.50 (2.80)	63.05 (5.12)	62.54 (4.96)	64.06 (5.28)	72.57 (4.62)	72.22 (4.44)	58.75 (5.46)
VAS sleepiness evening (%)	40.58 (4.29)	39.06 (3.83)	40.37 (3.85)	36.65 (5.01)	38.26 (4.18)	43.00 (4.82)	37.65 (4.42)
VAS sleepiness morning (%)	41.84 (4.28)	31.36 (3.73)	36.47 (4.29)	32.56 (4.27)	31.35 (3.81)	28.36 (3.45)	35.98 (4.77)
KSS sleepiness evening	6.09 (0.36)	5.79 (0.36)	5.68 (0.34)	5.72 (0.47)	6.00 (0.30)	5.79 (1.18)	6.14 (0.42)
KSS sleepiness morning	5.53 (0.40)	5.44 (0.39)	5.68 (0.35)	5.68 (0.40)	6.52 (0.31)	6.57 (0.33)	5.50 (0.44)

Mean values and standard errors (SE) are reported.

*BDC* baseline data collection, *HDT* head-down tilt, *R* recovery, *SWA* slow-wave activity, *ln* natural logarithm, *VAS* visual analogue scale, *KSS* Karolinska sleepiness scale.

^a^indicates significant differences to BDC-9 (only HDT1, HDT58, R + 1, and R + 12 were statistically compared to BDC-9, see Statistics).

**Fig. 2:**
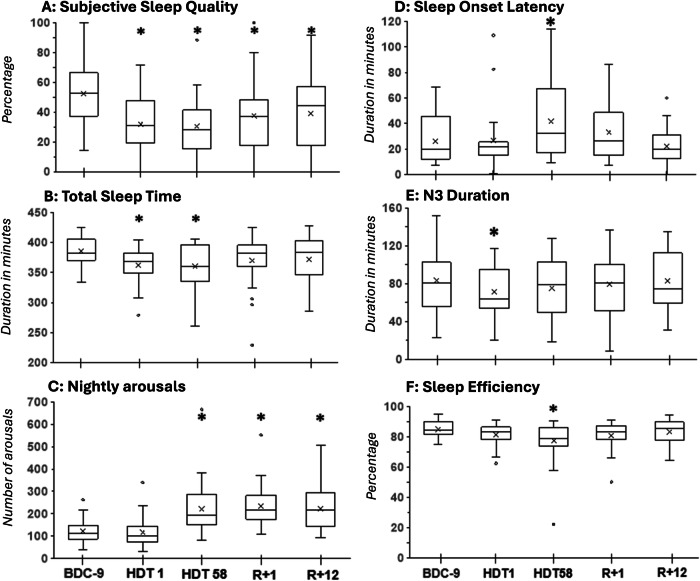
Objective and self-reported sleep parameters at baseline (BDC-9), head-down tilt (HDT) bed rest, and recovery (R). **A** Self-reported sleep quality, **B** total sleep time, **C** number of arousals, **D** sleep onset latency, **E** N3 duration, **F** sleep efficiency. Boxplots include mean values displayed as x. “*” marks a significant difference between HDT1, HDT58, R + 1 compared to BDC-9. R + 12 was only compared to BDC-9 in case R + 1 indicated a significant difference.

ANOVAs showed significant effects of condition for SPT, TST, SOL, sleep efficiency, N3, and the number of arousals/ arousal index, but not for TIB, N1, N2, REM, N1%, N2%, N3%, REM% and WASO. In comparison to the BDC-9, SPT and TST were decreased on HDT1 (SPT: *p* = 0.0168, TST: *p* = 0.0081) and HDT58 (SPT: *p* = 0.0009, TST: *p* = 0.0086). Arousals/ arousal index did not increase significantly on HDT1 (*p* = 0.4182/ *p* = 0.7907) but showed a significant increase on HDT58 (both *p* = 0.0004), R + 1 (both *p* = 0.0004), and R + 12 (both *p* = 0.0004). SOL was longer (*p* = 0.0207) and sleep efficiency poorer (*p* = 0.0150) on HDT58 compared to BDC-9. The duration of N3 was shorter (*p* = 0.0234) on HDT1 in comparison to BDC-9 (Fig. 2). ANOVA results did not reveal any sex differences (interaction all *p* > 0.1). Mixed ANOVAs on sleep efficiency (interaction *p* = 0.0027) and SOL (interaction *p* = 0.0001) showed significant effects of age. For SOL, post-hoc comparisons that were sliced by condition were non-significant. For sleep efficiency, post-hoc comparisons indicated significant differences at HDT58 and R + 1 which were in both cases attributable to one (but not the same) middle-aged individual. Thus, results did not indicate that the effects of HDT on sleep differed by sex and age.

**Fig. 3:**
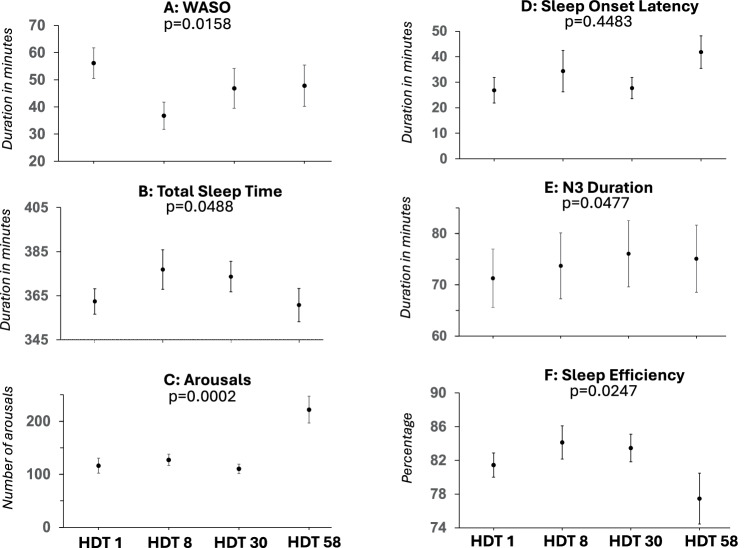
Sleep during 6° head-down tilt (HDT) bed rest. Mixed quadratic regression analyses of **A** *WASO* wake after sleep onset, **B** total sleep time, **C** number of arousals, **D** sleep onset latency, **E** N3 duration, **F** sleep efficiency. Mean values and standard errors are displayed.

Regression analyses of the HDT phase (Fig. 3) indicated a quadratic association between TST (*p* = 0.0488), sleep efficiency (*p* = 0.0247), arousals (*p* = 0.0002), arousal index (*p* < 0.0001), N3 (*p* = 0.0477), and WASO (*p* = 0.0158) with time spent in HDT. SOL did not show a time-dependent change (*p* > 0.15). In general terms, sleep improved during mid-HDT and deteriorated again at the end of HDT, in some cases (TST, SE, arousal) being worse than on HDT 1.

**Fig. 4:**
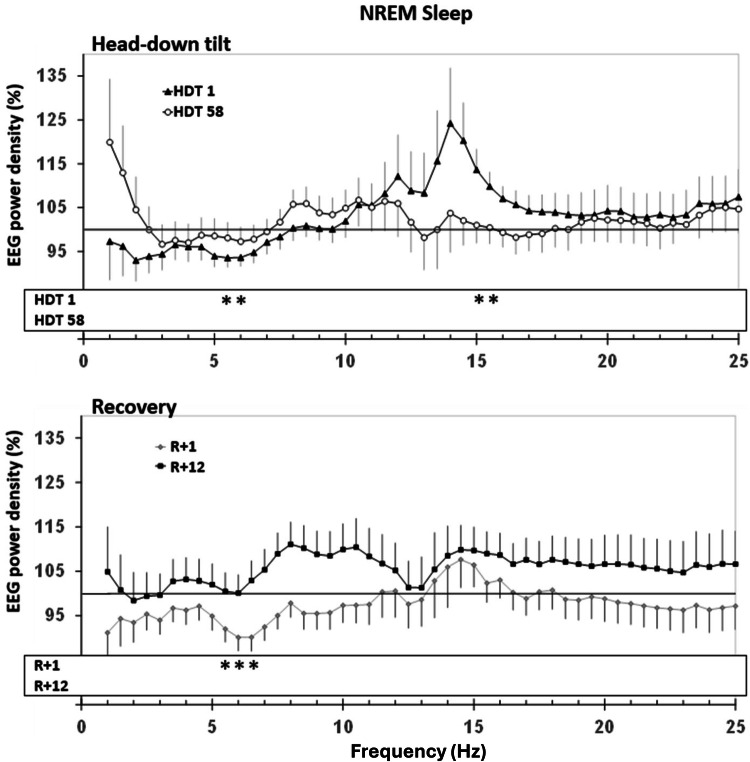
Relative sleep EEG power spectra for NREM sleep (N2 + N3) comparing 6° head-down tilt (HDT) bed rest and recovery (R) to baseline. Upper panel: Change in EEG power spectra in HDT1 and HDT58 from baseline. Lower panel: Change in EEG power spectra in R + 1 and R + 12. The probands‘ power spectra were referenced to his/her sleep at baseline (BDC-9, grey line at 100%) and then averaged within groups. Mean percentages and standard errors are displayed. Dependent *t*-tests were calculated to compare conditions to BDC-9. Significant differences that survived step-down Bonferroni adjustment within each frequency bin are indicated with stars “ *.” The lowest frequency bin was not included in the analyses due to the vulnerability to low-frequency artifacts.

**Fig. 5:**
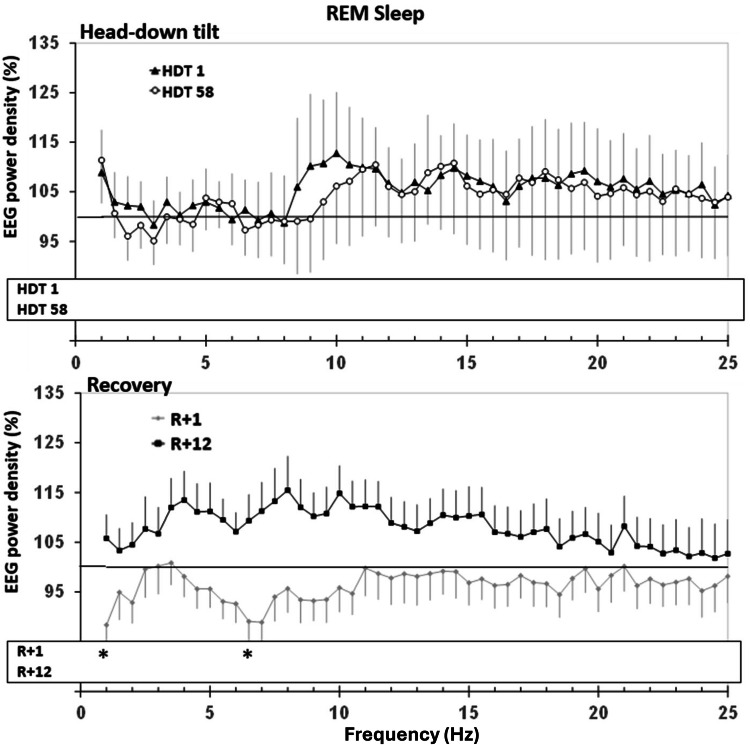
Relative sleep EEG power spectra for REM sleep comparing 6° head-down tilt (HDT) bed rest and recovery (R) to baseline. Upper panel: Change in EEG power spectra in HDT1 and HDT58 from baseline. Lower panel: Change in EEG power spectra in R + 1 and R + 12. For further explanation, see Fig. 4.

In comparison to BDC-9, spectral analyses indicated a decrease in EEG power density (Figs. 4 and 5) in lower frequencies on HDT1 and R + 1 during NREM sleep, as well as an increase in higher frequencies on HDT1. During REM sleep, power density was decreased on R + 1 at 1 Hz and at 6.5 Hz. SWA did not indicate significant differences.

### Self-reported sleep and sleepiness

The quality of sleep was evaluated as lower on HDT1 (*p* = 0.0003), HDT58 (*p* = 0.0003), R + 1 (*p* = 0.0094), and R + 12 (*p* = 0.0109) when compared to BDC-9. The perception of sleep need and recuperation after sleep as well as VAS and KSS sleepiness in the evening and morning, did not differ from BDC-9 during any of the tested nights (all *p* > 0.05). The self-reported quality of sleep correlated with N3 (*r* = 0.44, *p* = 0.0378). Subjective and objective SOL (*r* = 0.45, *p* = 0.063), and KSS sleepiness in the evening and N3 duration (*r* = 0.40, *p* = 0.065) correlated on trend-niveau. The remaining correlations were non-significant.

## Discussion

In the present study, we examined the effects of a 60-day 6^o^HDT bed rest in 24 male and female participants on objective and self-reported indices of sleep and sleepiness. Sleep was negatively affected by HDT; sleep duration, sleep efficiency, and N3 duration were reduced, SOL and number of arousals/arousal index were increased, and participants perceived their sleep quality as reduced. These effects on sleep were moderated by time spent in HDT. In the following, we focus on acute effects upon entry in HDT, long-term effects of continued HDT as well as on recovery from HDT.

Upon acute entrance into HDT, participants’ sleep duration was shorter and contained 12 min less N3 sleep. Both parameters are known to be essential for the recuperative value of sleep. This is in accordance with findings from polysomnographic measures on a shuttle mission^20^ and a study on the acute effects of 6° HDT bed rest, which reported a decrease in slow-wave sleep fraction^31^. In the present study, the decrease in sleep quality was also evident by reduced spectral power at some lower frequencies in NREM sleep. The acute deterioration of sleep quality was perceived and reported as a 20% decrease by the participants. However, they did not rate sleep as less recuperative and did not express an increase in sleep need or sleepiness. The latter was also observed by Komada and colleagues^31^. In line with this study, we also did not find an effect of HDT on light sleep (N1, N2) nor on REM sleep duration. However, in a study using 12° HDT bed rest, the fraction of light sleep increased, while N3 sleep and REM sleep were decreased^26^, indicating that the degree of HDT itself or the severity of the induced body fluid shift during bed rest is modifying the acute negative impact on sleep. The percentage composition of sleep stages in relation to TST, however, was maintained in our study. An acute increase in the number of arousals (short episodes of accelerated brain activity) has also been reported during 6° HDT^31^. While our study did not show such an effect, the changes in the EEG spectrum during NREM sleep (decrease of power at lower frequencies, increase of power at some frequencies above 14 Hz) have typically been associated with reduced sleep depth. Thus, EEG spectral analysis may provide a more sensitive measure of subtle changes in sleep depth and continuity associated with acute exposure to microgravity than the number of arousals.

After 58 days in HDT, sleep duration remained below the recommended sleep duration for adults^6^. It took participants 60% longer to fall asleep compared to baseline conditions. Sleep efficiency was reduced to 77% and sleep contained 80% more arousals than at baseline, a clear sign of sleep fragmentation. The effects on sleep efficiency and arousal seem a bit stronger than what is known from spaceflight, which might be explained by the different duration of HDT in our study compared to space missions. Even though participants still perceived sleep quality as reduced, this was no longer accompanied by a significant decrease in N3 duration or in spectral power of low frequencies. Indeed, Dijk and colleagues^5^—analysing short-term space missions—also reported subtle effects, i.e. a decrease of slow-wave sleep in-flight in the last third of the sleep episode only, while astronauts’ self-reported sleep quality was decreased and seemed to decrease (non-significantly) with time spent in-flight. Along these lines, Koller and colleagues^32^ found a decrease in the peak-to-peak amplitude of slow-waves, but not in slow-wave sleep duration per se. The significant quadratic association between sleep duration (TST), sleep efficiency, WASO, N3 duration, and number of arousals/ arousal index with time in HDT indicates that sleep duration and quality follow an inverted U-curve of acute negative effects upon entry in HDT followed by a short transient improvement to eventually a deterioration. This might explain why (apart from small sample sizes) studies that examined sleep in spaceflight or in simulated microgravity for up to four weeks did only observe subtle, if any, changes in sleep structure compared to pre-flight or baseline (on HDT21^33^, on night 10 and night 19 in dry immersion^34^, between nights 3 and 6, and nights 12 and 15^32^). Taken together, our results support the notion that sleep impairments during real or simulated microgravity do not abate over time.

On the second night after the end of bed rest (R + 1), most sleep parameters had already reverted to values not different from baseline. Yet, a decrease of EEG power in the low-frequency range during NREM sleep and still an elevated number of arousals/ arousal indexes hinted at incomplete recovery. A decrease in slow-wave sleep duration has been reported post-flight compared to pre-flight^5^, while the peak-to-peak amplitude of slow-waves was found to quickly renormalize after return to Earth^32^. We did not find a compensatory increase in REM duration, or a shorter REM latency as has been reported by Dijk and colleagues^5^. Astronauts on this shuttle mission lived on a sleep-wake schedule shorter than 24 h. Thus, they experienced a scheduled daily progressive phase advance in scheduled wake times. However, the authors deemed the observed REM changes more likely to be a non-circadian consequence of spaceflight rather than to be caused by the normalisation of the circadian phase angle upon return to Earth^5^. In the present study, on R + 12, the number of arousals/ arousal index was the only objective sleep parameter that was still elevated, equalling the numbers seen on HDT58, indicating a protracted recovery process. During recovery (R + 1 and R + 12), participants rated their sleep quality still worse compared to baseline. This is in contrast to post-flight data from shuttle missions in which astronauts assessed their sleep quality as better than pre-flight or in-flight^5^. Differences in the duration of exposure to HDT or space might be the reason for these divergent findings. Furthermore, the cognitive load of a space mission is very different than that needed during bed rest. Astronauts are used to extreme conditions and task loads, especially pre-flight. Post-flight schedules are after the work has been completed in orbit and are likely less stressful. Also, in contrast to astronauts who have returned home to Earth, family, and friends post-mission, our participants remained in the lab during recovery which might have differentially impacted self-reports.

It has been suggested that the reduced sleep duration of astronauts in space might be a consequence of social and operational demands or leisure activities that astronauts may prioritise over sleep^5^. As our study design used fixed light-dark cycles with sleep opportunities not changing over time, this cannot explain the reduced sleep duration observed in the present study. Subjects were not distracted by any obligations and had no social interaction possibilities during sleep time that could account for the significant shortening of sleep. Jones et al.^35^ postulated that stress might impact sleep duration. They conducted a longitudinal observational study on 24 astronauts to examine the association between sleep and ratings of stress and workload before, during and after 6-months ISS missions. They found that self-reported sleep duration of less than 6 h/night was associated with higher ratings of stress, and self-reported sleep duration of less than 7 h/night was correlated with lower subjective sleep quality. Those results might partly be transferable to our study, where self-reported sleep quality did not correlate with the objectively measured sleep duration but did correlate with N3 duration. Participants in our study might have perceived HDT1, HDT58, and R + 1 as slightly more stressful as more experimental examinations were scheduled compared to BDC-9 or HDT30. Results might also have been impacted by participants’ excitement at the end of HDT, though self-reported sleep depth was unchanged. Changes in circadian rhythmicity in space may impact sleep^19^. Even under lab conditions with constant light-dark and eating-fasting cycles, a small (< 30 min) circadian phase delay was reported after several weeks of 6° HDT^36^. It is possible—though not likely, given the present study design—that the observed sleep alterations in our study are due to a change in participants’ circadian phase angle. While all these abovementioned factors may contribute to poor sleep quality, they are probably not the main contributing reason in the present study. It seems more likely that the physiological changes induced by HDT (such as the fluid shift etc.) and the reduced physical activity play an important role. We did not find that HDT affected female and male participants differently or that the effects of HDT on sleep differed by age, although these findings have to be evaluated with caution due to the small sample size.

In order to promote longer sleep durations during space missions, some tools and countermeasures have been proposed, such as the implementation of stable schedules to minimise circadian misalignment or, if insufficient, the provision of sleep-promoting medication^16^. As we applied a stable sleep-wake cycle in our study, this countermeasure alone seems insufficient to prevent sleep from deteriorating. Light therapy is another promising countermeasure that has been successfully applied in treating circadian rhythm sleep disorders^37,38^ and in adapting circadian rhythms to a simulated Mars day^39^. The strategic use of caffeine was considered to improve performance^16^. These countermeasures could be tested in future ground-based studies.

Interestingly, there are studies that found an increase in slow-wave sleep in space^40–42^ and in HDT bed rest^43^. Gkivogkli and colleagues^43^ performed a long-duration 6^o^ HDT bed rest study of eight weeks and measured sleep with polysomnography over five nights. A control group was subjected to bed without intervention, whereas a training group was subjected to a reactive sledge jump training. Preliminary findings from that study suggest that while N3 duration decreased in the control group along with a decrease in sleep duration and REM duration, the training group showed an increase in N3 duration with preserved sleep and REM duration^43^. Similarly, an increase in slow-wave sleep duration has been reported under high workload conditions in cosmonauts during short- and long-term space flights on board of MIR space station^42^. The increase was also seen for the high workload situation that astronauts faced during Skylab I and II missions^40,41^ in which they conducted experiments, extravehicular activities, and Skylab repair. Based on these results, it may be worthwhile to further determine the extent to which a high physical workload and training can improve sleep quality. Thirty minutes of continuous or intermittent artificial gravity by centrifugation in our study were possibly too short in duration and not physically strenuous enough to improve sleep.

The results of the present study were obtained under highly controlled laboratory conditions. This is an advantage because it allows for excluding a variety of confounding factors. But at the same time, it is also a limitation, since it is unclear to what extent our results translate to real spaceflight conditions. As mentioned before, many factors likely contribute to sleep disturbances in space. They are in part caused by factors such as space motion sickness, perception of light flashes when high energy protons hit the retina, thermal discomfort or noise^19^, conditions that we choose not to simulate during bed rest studies. Still, bed rest represents an unprecedented model of standardised unloading that is less confounded by in-flight crew activities and is hence useful to better understand the mechanisms and rates of adaptation^22^ as a direct consequence of HDT bed rest. Although daily activities—required not to be activating—stopped at least two to three hours before PSG recordings, we cannot completely rule out that the testing during daytime impacted sleep. Sleep is affected by pain^44^ and particularly back pain, which often occurs in space^45,46^, but it also occurs during HDT bed rest^47^. Our subjects also reported pain in various parts of the body during and after HDT, which might have disturbed sleep. Respiratory disturbances and snoring have been reported to decrease in space^48^. In HDT bed rest, however, the fluid shift to the upper body is not accompanied by the unloading of the body through microgravity. Thus, it remains open whether sleep-disordered breathing contributed to the sleep disturbances that we observed in our study and should be tested in future studies.

With 24 participants who were examined with polysomnography before, during, and after HDT our sample size is large in comparison to most other studies. However, the sample size might have been too small to detect sex and age differences or to detect the effects of the applied countermeasures on sleep.

Sixty days of 6° HDT bed rest induced a substantial decrease in sleep duration and quality without indication of adaptive improvements during the course of the HDT bed rest phase. While sleep duration quickly recovered after reambulation, participants still experienced protracted sleep fragmentation. With regard to long-term space missions, astronauts should be made aware that sleep loss might accumulate over time, which could compromise optimal cognitive functioning^49^. Countermeasures for optimising sleep in space are needed.

## Data Availability

The datasets used and/or analysed during the current study are available from the corresponding author upon reasonable request.

## Code availability

The codes used for analyses of the current study are available from the corresponding author upon reasonable request.
